# Examining a Digital Health Approach for Advancing Schizophrenia Illness Self-Management and Provider Engagement: Protocol for a Feasibility Trial

**DOI:** 10.2196/24736

**Published:** 2021-01-25

**Authors:** Sean Kidd, Kwame McKenzie, Wei Wang, Sacha Agrawal, Aristotle Voineskos

**Affiliations:** 1 Department of Psychiatry University of Toronto Toronto, ON Canada; 2 Centre for Addiction and Mental Health Toronto, ON Canada

**Keywords:** schizophrenia, psychosis, digital health, mobile health, smartphone

## Abstract

**Background:**

In schizophrenia spectrum populations, adherence to treatment is poor, community-based supports are limited, and efforts to foster illness self-management have had limited success. These challenges contribute to frequent, lengthy, and costly hospital readmissions and poor functional outcomes. Digital health strategies, in turn, hold considerable promise in the effort to address these problems.

**Objective:**

This feasibility trial will examine a digital health platform called App4Independence (A4i), which was designed to enhance illness self-management and treatment engagement for individuals with schizophrenia.

**Methods:**

Feasibility metrics in this single-blind, randomized trial include study recruitment and retention, rate of technology use, safety, and utility in clinical interactions. Other outcome metrics include symptomatology, treatment adherence, patient-provider alliance, and quality of life. In this trial, 160 study participants with schizophrenia spectrum diagnoses will be randomized to either treatment or control conditions, with pretest-posttest outcomes measured over a 6-month period.

**Results:**

This study was funded by the Canadian Institutes of Health Research in January 2020 and received Institutional Review Board approval on August 13, 2020. This study plans to begin recruiting in January 2021 and will be completed within 3 years. Data collection is projected to begin in January 2021.

**Conclusions:**

This research will provide critical information for the development of this new technology in the larger effort to address a key problem in the schizophrenia field—how to leverage technology to enhance illness self-management and care engagement in resource-limited service contexts.

**International Registered Report Identifier (IRRID):**

PRR1-10.2196/24736

## Introduction

Advancing digital health interventions in schizophrenia is important in the Canadian health care context. Schizophrenia is responsible for 3.8% of the hospital admissions in Canada and accounts for an estimated annual cost of Can $6.85 billion annually in health care and lost productivity [1]. The associated challenges include high rates of completed suicide, low quality of life, poor access to nonpharmacological interventions, and pharmacological interventions that have suboptimal effects [2]. Overall, schizophrenia has proven extremely difficult to treat as is evidenced by high relapse and readmission rates [1]. The most common contributors to relapse in schizophrenia are medication nonadherence, substance use disorders, social isolation, and inadequate supports [3-6].

There has been extensive commentary on the system of care problems for people with schizophrenia. Within these systems, the primary point of engagement—interactions and relationships with health care providers—is challenged. In inpatient units, patients are frequently dissatisfied with custodial approaches to care [7,8], and in outpatient settings, the frequency and nature of contacts with providers are often inadequate. Common concerns include a lack of shared-decision making in treatment, limited support in illness self-management, and insufficient time with providers [5,9,10]. Such suboptimal provider engagement and outpatient support have been directly implicated in treatment adherence rates of 50% or less [11] and poor quality of life [12,13]. In this context, leveraging technology to enhance engagement in outpatient care and to provide support with illness self-management holds considerable promise to (1) ameliorate aspects of schizophrenia that challenge provider contacts (eg, cognition, anxiety) [14,15], (2) facilitate provider access to more detailed information from which to base care decisions, (3) lead to patients feeling more empowered in the care process [16], and (4) lead to enhanced treatment engagement with implications for lower relapse rates [5,10,17]. Furthermore, digital engagement can facilitate more frequent supportive contacts at much less expense than in-person contacts and can offset challenges such as travel time and expense and the stigma of mental illness that can make some individuals reluctant to pursue care in psychiatric services.

To date, evidence for technology-enabled approaches to enhance outcomes for individuals with severe mental illnesses is limited. In particular, feasibility has proven to be a major challenge. In-office technologies require space, infrastructure, and staffing, and have proven difficult to implement due to expenses [18,19]. Web-based approaches are less expensive but have demonstrated high rates of attrition [20]. There are very few mobile apps targeting schizophrenia, in contrast with the large volume of apps for conditions such as anxiety and depression [21]. The reasons for such a gap are unclear, given the observation that over 80% of individuals with schizophrenia and other psychoses routinely use mobile technology and the majority are interested in using technology to assist with illness management [22-25].

A number of pilot studies suggest that schizophrenia-targeted mobile and web-based apps in areas such as cognitive remediation are feasible and do not result in any noted risks [23,26]. Most directly relevant to this proposal is the work of Ben-Zeev and colleagues [27]. Their FOCUS app has features that include daily activity prompts, brief self-assessments, and coping strategy tips. Preliminary investigation of this app indicated no risks associated with its use and sustained use over several months. Another similar digital health approach in this area that has shown feasibility is PRIME, which was developed by Schlosser and colleagues [28]. The core function of PRIME is providing users with access to masters-level clinicians who provide strategy coaching. There is, however, a paucity of trial data. In one of the few examples, the PRIME app described above was recently trialed [29] and demonstrated, compared to treatment-as-usual (TAU), improvement in some social engagement and depression metrics though no changes in psychosis symptoms, quality of life, or functioning were observed. Systematic reviews [30,31] have highlighted the need for more clinical trials in this area.

In this initial pilot, we focused on the early-stage testing of a digital health tool, App4Independence (A4i), which was performed in 2 steps. Following a preliminary 1-week beta test by 5 individuals to address technical issues, the qualitative and quantitative A4i outcome data from 38 individuals were analyzed, assessing feasibility over a 1-month period [32]. The mean number of interactions with the app per day was 4.21 and, by day 20, a 4% churn rate was observed (rates of individuals who ceased app use). Considering outcomes (noting the lack of a control group), small-to-medium effect changes were observed in several symptom domains: a significant decrease in the depression domain of the Brief Symptom Inventory with a medium effect size (ES=0.42) was observed, along with decreases in paranoid ideation (ES=0.29), psychoticism (ES=0.22), obsessive compulsive symptoms (ES=0.38), phobic anxiety (ES=0.38), and interpersonal sensitivity (ES=0.18) with depression, obsessive compulsive, and paranoid ideation findings being the most robust. Signals of improvement were also seen in medication adherence and ratings of personal recovery, though changes in these areas were limited. Frequency of use did not appear to be related to outcomes, though those who used the app more frequently were more unwell in several symptom areas, including depression. With disagree, neutral, and agree options on satisfaction scale items, the mean average “agree” response was 68% (with agree being positive). Qualitative feedback was primarily positive: “easily reminding me about the next time I need to take my meds;” “(it helps me) redefine my daily thoughts…for people to feel mentally healthy;” and “helps you focus on something when your thoughts are racing.” Critical feedback was largely in the realm of minor enhancements. Only 1 participant noted that the texts made him/her anxious though they also noted that they would “definitely” use A4i in the future, if available.

The trial described in this protocol is the next step in the program of work surrounding A4i—moving on to a feasibility randomized trial. Although this trial is in an early stage, it is one of the most rigorous trials conducted to date of a digital health approach tailored to schizophrenia. This test is needed because (1) this is a rapidly advancing area in which there are multiple calls for better feasibility and effectiveness data, (2) if ultimately effective, A4i will provide a cost-effective approach for a major health care problem, and (3) this study is an important step for the development of A4i, now that we have pilot data for our prototype.

## Methods

### Trial Design

This feasibility study [33,34] will employ a 2-arm, randomized controlled trial design. The design will be single blind as participants will be aware of A4i exposure with the assessor blinded. Measures will be completed by both care providers and persons receiving care, with the latter being adults with schizophrenia spectrum diagnoses receiving care in a large diverse Canadian city.

This test will determine the following 2 issues:

1. Progression criteria, that is, first, meeting the recruitment target and assessing recruitment rate considerations that may be unique to hospital and community service sites and achieving a representative sample, and second, obtaining outcome data for at least 80% of those recruited. An 80% target would seem indicated, given the retention in the small number of previous studies ranging from 88% (3 months of technology use, PRIME) to 94% (6 months of technology use, FOCUS). Shorter tests have yielded retention closer to 100% (eg, the 1 month pilot of A4i). The third criterion is sustained use of A4i for an average of at least 75% of the weeks in which it was installed (based on a finding of 80% for FOCUS in a 6-month test period), and the fourth criterion is a lack of emergence of significant safety concerns. The fifth criterion is the patient and provider satisfaction with using the provider portal in clinical contacts.
2. Preliminary outcome data in domains hypothesized to be relevant to the likely effects of A4i, including symptomatology, treatment engagement, clinical alliance, and quality of life, as compared with TAU. These data will help to inform and refine A4i treatment target hypotheses and assist with sample size determination and outcome timeframes for effectiveness trials.

### Trial Interventions

The treatment condition is 6 months of participants being provided with A4i. A4i was developed by the Centre for Addiction and Mental Health (CAMH, Academic Health Sciences Centre in Toronto, Canada) and the Canadian digital health company MEMOTEXT*.* An iterative design and development model [35] was employed to determine the requirements of A4i.

Stage 1: Literature, patent, and commercial market reviews.

Stage 2*:* Focus groups with patients, family, psychiatrists, and case managers.

Stage 3: An initial test of a beta version by 5 individuals with psychosis for 1 week.

Stage 4: Review of initial test findings and iteration of app design.

Stage 5: Pilot testing by 38 individuals over a 1-month period [32] followed by further iteration. Specific modifications following the pilot study included enhancing the general ease of use of all features, modifications to the auditory hallucination detector to allow accuracy ratings, and modifications to enhance the care provider interface and the quality of the summaries for clinicians.

**Figure 1:**
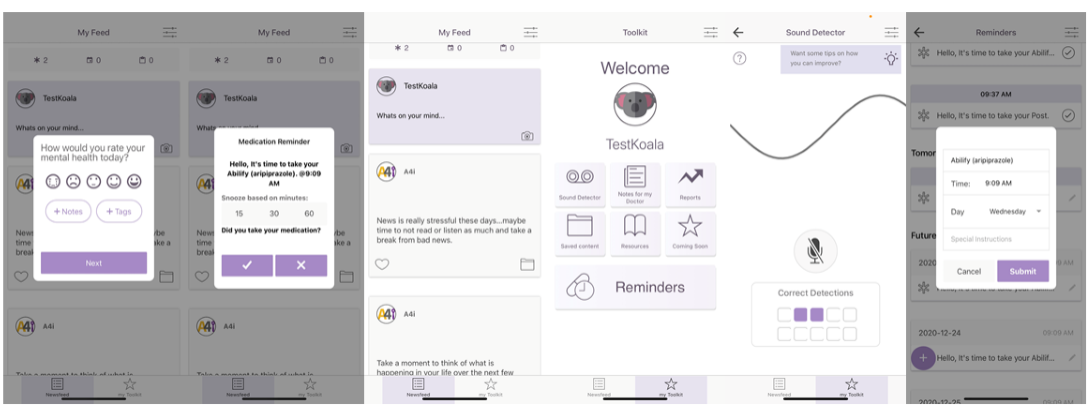
A4i screenshots.

**Figure 2:**
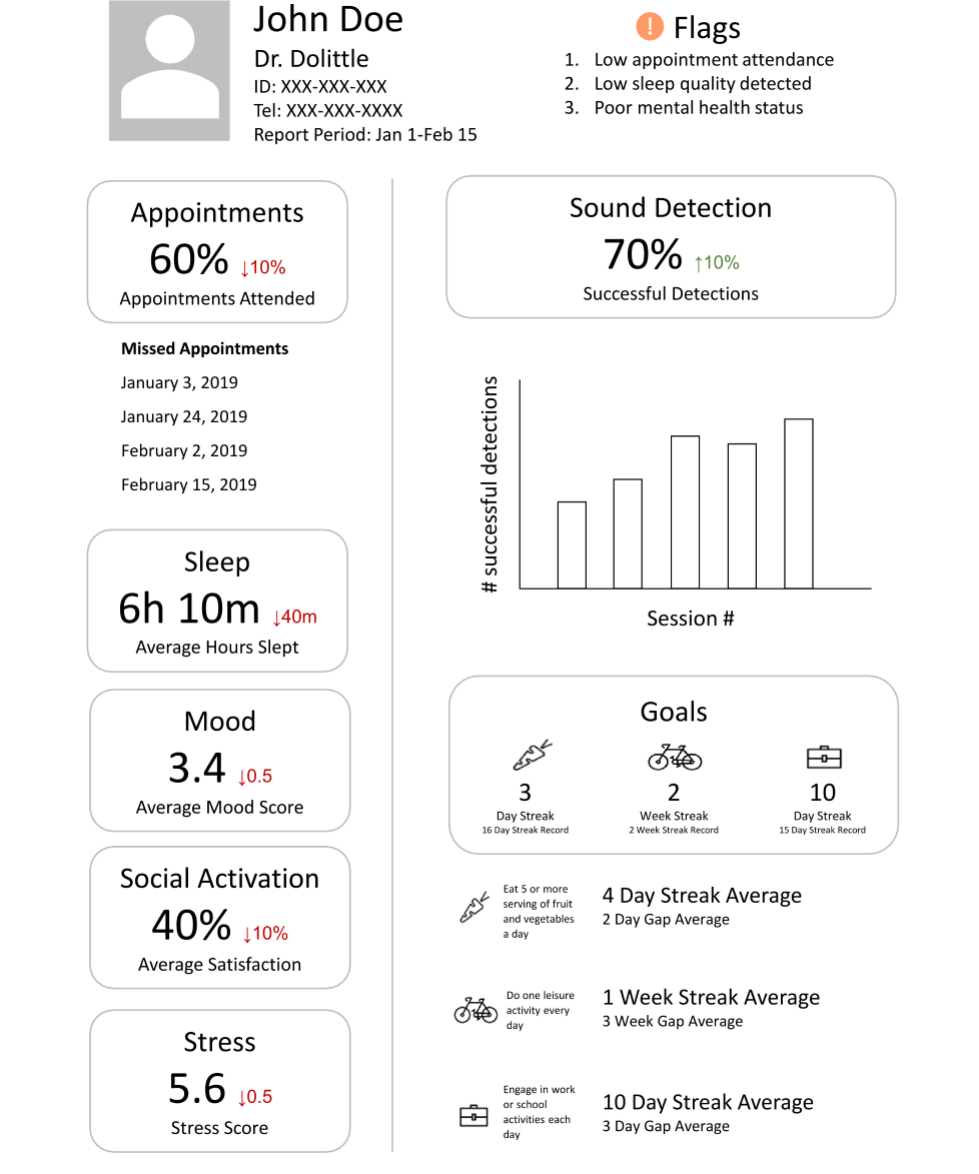
Health care provider's dashboard.

A4i works on both Android and iPhone platforms, for those with and without data plans (those who rely on Wi-Fi), and is in a testing phase and not yet publicly available. Specific A4i functionality includes (see Figure 1, Figure 2, and a demo [36]) the following:

1. Addressing social isolation and cognitive challenges through personalized prompts and the scheduling of activities. These events are entered into a scheduler, with prompts texted through a feed and self-report data collected on attendance (for events) and adherence (for medication reminders).
2. Fostering illness self-management through evidence-informed content that makes suggestions and provides resources relevant to coping with psychosis symptoms, negative symptoms of schizophrenia, cognitive challenges, motivation, and anxiety. The content concentrations are determined by an algorithm built from a short screener completed at the time of upload. This information is also delivered through the feed with the ability to bookmark favorite items into a personal library.
3. The trial intervention includes a peer-peer engagement platform that facilitates strategy/tip sharing between users (anonymous and moderated). This aspect of the platform also functions through the feed.
4. Daily wellness and goal attainment check-ins to highlight mental health trajectories gathered through self-report on rating scales that are prompted daily. This information is graphically summarized for review by the user with goals entered at onboarding and is modifiable.
5. An ambient sound detector with an oscilloscope-type indicator that assists individuals with auditory hallucinations in the effort to separate hallucinations from real sounds with users able to rate and track their responses in terms of success with discernment. Similarly, use and outcome ratings can be reviewed by the user.
6. Passively collected data on phone use as a proxy for sleep and activity levels (generally, when people are awake, they are using and moving their phones) that in the future may prove helpful in developing predictive analytics regarding relapse.
7. A provider dashboard that, with appropriate consent, is provided to the individual’s provider prior to their appointment (for this trial, the dashboard was provided via study staff; in future, it will be accessible via electronic medical records). This was co-designed with psychiatrists and patients and generates a summary of day-to-day wellness ratings, responses to reminders, goal progress, sleep detection, self-reported medication adherence, and a “notes for my provider” function (eg, “Please can we discuss my medications making my mouth very dry”).

In the treatment condition, A4i will be installed on participant phones for 6 months following screening and baseline assessment completion. Should participants show no interactions with the app in the first 2 weeks or respond to an app prompt regarding A4i utility that there is a problem, they will receive a phone call from a research assistant uninvolved in assessments to coach through and troubleshoot challenges. Both control and experimental condition participants will be receiving standard outpatient care (TAU) for psychosis involving routine (at least monthly) contacts with a care provider. The centralized recruitment process at CAMH facilitates tracking and control over participation in other treatment studies that may confound the findings. For community sites, participants will be asked about their involvement in any other trial (less likely than CAMH) with study implementation adjusted to address potential confounds. Note that those assigned to TAU will be provided with access to A4i after their 6-month evaluation for a period of 6 months if they are interested in this option.

### Randomization and Addressing Bias

A 1:1 blocked randomization, stratified by gender, will be employed to ensure balance in sample size between treatment and control groups and gender representation. Stratification by study site (CAMH vs community agency) will also be undertaken to determine feasibility questions that may differ as a function of same. REDCap (Research Electronic Data Capture) will be employed and will allocate based on the computer-generated randomization list. Allocation concealment will be achieved since the person making the assignment will have no awareness or control over the randomization schedule. Comparison with TAU is single blind as it is not possible for participants to have adequate information to consent without knowing that they are or are not using the technology. Randomization will be secure as noted above. Statistical analyses will be performed by a biostatistician uninvolved in study operations and kept blind to treatment conditions. During all operational meetings, steps will be taken to ensure that participant identifiers are managed such that the risk of unblinding to assessors is minimized. If unintended unblinding occurs, the event will be documented and arrangements made for a blinded postassessment. Assessors are masters level, with interrater reliability established through mock cases and meeting a minimum of 80%. Finally, participants will be asked to not discuss with the assessor whether they used A4i or not.

### Inclusion/Exclusion Criteria

#### Inclusion Criteria (Participants With Schizophrenia)

Participants will be adults, 18 years of age or older, with a chart diagnosis of a Diagnostic and Statistical Manual of Mental Disorders, Fifth Edition (DSM-5) schizophrenia spectrum illness (schizophrenia, schizoaffective, schizophreniform, delusional disorder, brief psychotic disorder, unspecified) confirmed by a structured diagnostic interview (SCID-5) [37]. All participants will be engaged in outpatient psychiatric treatment. They need to be proficient in English and own and use an Android phone or iPhone.

#### Exclusion Criteria

Participants will be excluded if they lack the capacity with no identified substitute decision maker or if they have an intellectual disability.

### Criteria (Service Providers)

Service providers will be psychiatrists and case managers engaged in the care of the participants.

### Measures

For the purposes of this trial, only pretest-posttest (baseline to 6 months) measures will be completed. It is also of note that follow-up data are less relevant for digital health approaches for which ongoing use is anticipated rather than clear end dates as are in the case with other clinical interventions. Measures will be completed virtually or in-person, with an emphasis initially on virtual assessment due to pandemic circumstances.

#### Feasibility Indicators

1. Assessment of numbers approached, successfully screened, consented, and numbers assessed at both time points to evaluate recruitment and retention. Additionally, recruitment rates will be compared between CAMH and collaborating community sites.
2. Objective A4i use metrics will be collected, including information on the time, frequency, and nature of use for each participant.
3. An unblinded research assistant will contact treatment arm providers and participants to complete a brief semistructured interview assessing strengths (generally and in clinical interactions) and limitations of A4i and any risks not otherwise reported or observed during study operations. Use of A4i-generated summaries for providers will be assessed both in terms of provider and participant reports of quality in clinical interactions and frequency and duration of use in a given clinical interaction. Patient satisfaction will also be examined with the 26-item scale used by Ben-Zeev and colleagues [26].
4. Safety will be assessed through information gathered via all study-related interactions with significant safety concerns operationalized as one or more critical incidents occurring in which there is evidence of an association between the incident and A4i use. Worsening in one or more outcome areas as compared with TAU would be another domain that would be flagged.

#### Clinical Outcome Metrics

1. Given the observation of improvement in psychiatric symptomatology in pilot testing and the importance of symptom severity for the quality of life and other key outcomes for people with schizophrenia [38], symptom severity is considered the primary outcome. General symptomatology will be assessed using the 53-item, 5-point Likert scale Brief Symptom Inventory [39]. Schizophrenia-specific symptomatology will be assessed with the Positive and Negative Syndrome Scale [40] with negative symptoms assessed with the Scale for the Assessment of Negative Symptoms [41].
2. Treatment Engagement was not fulsomely tested in the A4i pilot study due to its short duration, though it is of critical importance to considerations such as rehospitalization [3]. Accordingly, treatment adherence will be measured using (1) the 4-item Brief Adherence Rating Scale [42] with responses obtained by both providers and participants to assess medication adherence, (2) the 5-item, 6-point Likert scale Medical Outcomes Study general adherence scale [43] to capture broader adherence to treatment recommendations (again triangulated with provider responses), and (3) the percentage of scheduled appointments attended through electronic medical record audit at CAMH or provider report at non-CAMH sites. This broader approach to assessing adherence is necessary, as it is a construct that captures both medication regimen adherence and care team engagement.
3. Provider-Patient Clinical Alliance will be assessed with STAR (Scale to Assess Relationships) [44]. This 12-item measure employing a 5-point Likert scale has been used extensively in studies of outpatient care for severe mental illness. This measure supports patient and provider versions.
4. The Heinrichs-Carpenter Quality of Life Scale, which has 21 items, is well validated for schizophrenia [45] and captures sense of purpose, motivation, emotional, and social interaction, role functioning, and engagement in regular activities.
5. Descriptive Measures include core demographics (ethnicity, sexual orientation, age, education, etc, assessed at time 1 and service use history (hospitalization) assessed at both time points and triangulated by providers and participants with schizophrenia. Gender, specifically, will be determined through baseline self-report of female, male, transgender (male-female or female-male), nonbinary, or other. Medication delivery method (injectable vs oral) will be assessed before and after the intervention as this has implications for tracking medication adherence.

### Sample Size and Recruitment

We propose to recruit 160 participants, that is, 80 per group. The sample size determination was guided by 2 underlying power considerations driven by our aims. First, we expect to establish the feasibility of the trial by obtaining reliable estimates of feasibility indicators, for example, retention rate and completion rate of key measurements [46]. With 160 participants, we would achieve a small margin of error of 2.8% for an expected retention rate (85%). The margin of error increases to 3.7% for estimating the minimum proposed completion rate of measurement (expected at 80%) with attrition taken into account. Second, while we do not anticipate to have full power to detect treatment effect compared to TAU, given the feasibility test objective of this trial, the proposed sample size will give us a reasonable chance (64%) to detect a small-to-moderate effect (ES=0.40). The small-to-moderate effect size is in line with some of the findings in the pilot study, with the caveat that the pilot was an uncontrolled test of outcome. With longer treatment period, we expect to see larger effects as well. The power calculation assumed a level of significance of .05 and accounted for 15% attrition. Recruitment will occur through 2 primary sources. First, recruitment will occur through a centralized CAMH referral process located in the psychosis early intervention service and advertisements and contacts with clinicians in other CAMH schizophrenia services. Across these 2 sets of CAMH services, approximately 3000-4000 patients are annually registered. In the pilot study of 1 month of use (not including an early beta test, recruitment period was September 2017-March 2018), the centralized early psychosis recruitment process was the primary referral route and yielded a rate of 5.5 study completers/month. In the proposed trial, along with this centralized early intervention recruitment process, a greater emphasis will also be placed on recruitment through general schizophrenia services at CAMH. The second major source of recruitment will be community provider sites in Toronto. These 3 sites, all with high rates of contact with the target population, are Canadian Mental Health Association (Toronto), the Schizophrenia Society of Ontario, and Progress Place. At community sites, engagement strategies will include presentations by the principal investigator and research staff, posters, and introductions to clients by providers oriented to A4i. Key differences between the pilot and this trial are the longer duration of A4i use (6 months vs 1 month), which may slow recruitment, and the longer duration of the study which, for some aspects of the strategy, may improve recruitment as providers become better oriented and refer more routinely. Accordingly, it is conservatively estimated that we should be able to obtain a recruitment rate of 7-8 participants per month on average. Recruitment would occur over a period of 21 months.

### Compliance and Attrition

Engagement in the use of A4i once uploaded is expected to be good. In our feasibility testing, out of 38 users, 2 (primarily attributed to forgetfulness) could be considered noncompliant with minimal app use over a 1-month period. This is consistent with findings from Ben-Zeev’s group [26,27]. To define compliance, the rolling retention and churn rates of app usage were considered with 2 of the 38 users not returning to the app up to or after 20 days of use. However, it is to be expected that this might be higher over a 6-month period. App features that assist with engagement include the personalization of the peer-to-peer social feed based on the user’s intake profile, daily wellness check-ins that are accompanied by motivational content, an escalation of medication and appointment in-app reminders where the reminder will be delivered directly through SMS if no response is provided within an hour of receiving it, and provider engagement about the app. As well, as noted, indication of initial challenges with app use will prompt a call from a research assistant, which might assist with engagement. We experienced no loss to follow up in feasibility testing. This is similar to other studies in this area [27,28] though, again, this will probably be higher over a 6-month period; hence, our attrition estimate of 15%.

### Safety Considerations

None of the published works in the area of digital health approaches for schizophrenia has identified significant user risk. The only potential exception that we found was that a user of FOCUS became “paranoid about his mobile phone and broke it.” We have not experienced any incidents of concern in our pilot testing of A4i. However, we have been cognizant of the potential risks. For example, there was an instance of our moderator of the peer-peer strategy network intercepting a post that might be a problem (incoherent content and statement about ceasing medication). Our response was to reach out to the individual to discuss the post and assess the risks (no association with A4i use was indicated) and inform the individual’s care provider with participants’ consent. Participant privacy is another essential consideration. This risk is mitigated through tricouncil protocols for data safety and storage and MEMOTEXT only having access to phone numbers and provider dashboard data, with that information stored and managed in compliance with provincial and federal data safety and privacy requirements and approved by the CAMH Privacy Officer and Research Legal. Specific protections include the use of duplicate networks and backups, web application programming interfaces, and secure file transfer protocols, where files are encrypted with PGP keys, and only internal personnel may access data from secured locations with 2-level password access and Microsoft Security Best Practices followed regarding password complexity and a 90-day expiration cycle. Further information can be found in the pilot study report [32].

### Data Analysis

The data analysis strategy is as follows:

1. A4i use and satisfaction, along with feasibility metrics, will be examined descriptively with comparisons by gender, age, and study site (CAMH vs community) completed using nonparametric Kruskal Wallis H test (continuous variables) and chi-square analysis (categorical variables) to detect differences.
2. Qualitative data collected from A4i users and providers will be analyzed using qualitative content analysis procedures [47].
3. Descriptive statistics will be used to summarize the data on all participants to understand the unidimensional and multidimensional characteristics of data distribution and confirm the balance between the 2 groups. To evaluate the treatment effects on the primary and secondary outcomes, we will employ the intent-to-treat approach and use generalized linear models as the primary analytic approach, of which the baseline to 6-month change score will be treated as the response variable and treatment assignment, regardless of compliance, as the primary predictor with the corresponding baseline outcome measure, key demographic variables, and study site being controlled as covariates. The generalized linear model could accommodate potential deviation from normality of the outcome variables. The multiple imputation method [48] will be used to handle missing responses and account for potential bias. Three additional analyses will be conducted in addition to the primary analysis. First, we will conduct a sensitivity analysis by including the number of contacts with the psychiatrist in the model as an additional covariate to be controlled. Second, we will explore the moderation of the treatment effect of baseline symptom severity and key demographic variables by adding their interaction with the treatment assignment in the model. This may provide suggestive evidence of differentiated treatment effects. Third, we will look at the impact of compliance by correlating the change score of the outcomes with the app use for the subjects under the treatment condition. Additionally, if more than 10% of the subjects show no app use, a sensitivity analysis will be conducted with only subjects who used the app at least once. We will complete the analysis upon completion of the postintervention assessments. Given the possible importance of gender (female, male, and nonbinary) and age moderation factors, we will test if they have any effect on the magnitude of change in the outcomes. This will be done by adding an interaction between gender and treatment assignment indictor to the model described above.

## Results

This study was funded by the Canadian Institutes of Health Research in January 2020 and received Institutional Review Board approval on August 13, 2020. The anticipated recruitment start date for this trial is January 2021. However, the global pandemic has caused some delay in the initiation of the trial and may continue to cause delays depending on how the second wave affects the recruitment sites.

## Discussion

This trial is needed now as it will make a significant contribution to the evidence in this emerging area and it is a test of a unique technology. Given the high relapse rates among populations with schizophrenia and the Can $1000+/day cost of inpatient care, even modest effects of a technology such as A4i are of note, given its relatively low expense and ready access. If successful, technologies such as A4i might reduce the reliance on in-person treatment and augment and enhance the quality of in-person treatment and create access to supports for those who have situational (eg, rural) or other challenges (eg, motivation, self-stigma) that impede access to standard care. At the systems level, should the promise of early risk detection and mitigation be realized, the reliance upon costly, crisis responses may also be positively affected. However, such business cases will rely upon rigorous lines of investigation of which A4i is one example. Unfortunately, research rigor in digital health is more the exception than the rule at this stage of the field. Such work is essential in the larger effort to ensure that the public and service providers are not misled by unsubstantiated claims of effectiveness. Lastly, this work will bring important information forward to broaden the conversation about measurement-based care in psychiatry. Specifically, platforms such as A4i afford the opportunity to provide more frequent and nuanced assessments of symptomatology and functioning. Such information might prove important in refining medical and behavioral care pathways and algorithms to optimize the outcomes of those with complex conditions such as schizophrenia.

This trial will provide information that will be critical in determining if this technology is ready to move on to an effectiveness trial or if further iterations are needed. With these requirements established, future trials would move on to examine (1) effects observations with larger samples, (2) comparison with a sham condition such as a generic wellness app, and (3) sustainment of use and gains over longer periods. Knowledge exchange activities will include (1) publication in a relevant academic journal, (2) presentation in at least one international conference (eg, Schizophrenia International Research Society Conference) and one eHealth industry conference (BIO), and (3) a webinar advertised through research, practice, and administration networks (eg, Health Quality Ontario, Council of Academic Hospitals of Ontario; Orygen-Australia; RAISE trial network-United States).

## Appendix

Multimedia Appendix 1 Grant reviews report.

## Abbreviations

A4i: App4Independence
CAMH: Centre for Addiction and Mental Health
DSM-5: Diagnostic and Statistical Manual of Mental Disorders, Fifth Edition
ES: effect size
TAU: treatment-as-usual
