# Supplementary material for: Examining a Digital Health Approach for Advancing Schizophrenia Illness Self-Management and Provider Engagement: Protocol for a Feasibility Trial
Source: JMIR Res Protoc. 2021 Jan 25;10(1):e24736. doi: 10.2196/24736 (PMC7870355; doi:10.2196/24736)
Supplement: Multimedia Appendix 1 [file resprot_v10i1e24736_app1.pdf]

**Review Type / Type d'évaluation:**	Reviewer 1 / Évaluateur 1
**Name of Applicant / Nom du chercheur:**	Kidd, Sean
**Application No. / Numéro de demande:**	426889
**Agency / Agence:**	CIHR/IRSC
**Competition / Concours:**	Project Grant/Subvention Projet
**Committee / Comité:**	Randomized Controlled Trials 2/Essais contrôlés randomisés 2
**Title / Titre:**	Examining a digital health approach for advancing schizophrenia illness self-management and provider engagement

Adjudication Criteria/Critères de sélection

Significance and Impact of the Research/Importance et impact de la recherche: 4.2

Approaches and Methods/Approches et méthodes: 4.2

Expertise, Experience and Resources/Expertise, expérience et ressources: 4.2

Top/Bottom Selection/Groupe supérieur/inférieur

- (●) **Top/Groupe supérieur**
- ( ) **Bottom/Groupe inférieur**

**Review Type / Type d'évaluation:**	Reviewer 1 / Évaluateur 1
**Name of Applicant / Nom du chercheur:**	Kidd, Sean
**Application No. / Numéro de demande:**	426889
**Agency / Agence:**	CIHR/IRSC
**Competition / Concours:**	Project Grant/Subvention Projet
**Committee / Comité:**	Randomized Controlled Trials 2/Essais contrôlés randomisés 2
**Title / Titre:**	Examining a digital health approach for advancing schizophrenia illness self-management and provider engagement

Summary of Application/Résumé de la demande:

•Schizophrenia relapse is a significant cause of hospital admission in Canada

•The most common contributors to relapse in schizophrenia are medication non-adherence, social isolation, and inadequate supports

•>80% of individuals with schizophrenia routinely use mobile technology and are interested in using technology to assist with illness management; however, there are few examples of mobile applications targeting schizophrenia

•A4i app has been beta tested and many unique features (e.g., addresses social isolation and cognitive challenges through prompts, enables scheduling of activities, connects users to resources, enables illness self-management, has anonymous moderated peer-support platform, has daily wellness and goal “check-ins”, has an ambient sound detector to assist individuals with separating hallucinations from real sounds)

•The investigators propose a single-blinded, multi-site (n=4) RCT to test the feasibility and estimate effect of a digital health platform for improving clinical outcomes and care engagement for individuals with schizophrenia

**Review Type / Type d'évaluation:**	Reviewer 1 / Évaluateur 1
**Name of Applicant / Nom du chercheur:**	Kidd, Sean
**Application No. / Numéro de demande:**	426889
**Agency / Agence:**	CIHR/IRSC
**Competition / Concours:**	Project Grant/Subvention Projet
**Committee / Comité:**	Randomized Controlled Trials 2/Essais contrôlés randomisés 2
**Title / Titre:**	Examining a digital health approach for advancing schizophrenia illness self-management and provider engagement

Strengths and Weaknesses/Forces et faiblesses:

Strengths:

• Important issue - important population

•Strong team including clinicians, scientists, a biostatistician and technology collaborators

•PI is senior-scientist, well-published, focused on marginality and service enhancement for homeless and individuals with SMI

•Feasibility indicators are good

•Clinical outcomes are good

•Arms length DSMB

•Analyses by gender being planned

•Biostatistician performing analyses will be blinded to treatment allocation

Weaknesses:

•It states that double-blinding won't be possible; however, it may be possible that TAU participants will be dissatisfied with group allocation if they learn of the A4i intervention. Could one of the following two strategies be employed?

oA double-blinded study where inclusion for all requires a smartphone, and where potential participants are told they will be randomized to one of two interventions but not given specific detail as to what the interventions are. Or,

oA waitlist approach be used, where TAU participants could be offered use of the digital health platform after 6 month follow-up?

•Certain self-reported outcomes looking back over 6 months, like medication adherence and hospitalizations, may be limited due to recall bias

•Client volumes for community sites are not provided, although it is mentioned they have high rates of contact with the target population

**Review Type / Type d'évaluation:**	Reviewer 1 / Évaluateur 1
**Name of Applicant / Nom du chercheur:**	Kidd, Sean
**Application No. / Numéro de demande:**	426889
**Agency / Agence:**	CIHR/IRSC
**Competition / Concours:**	Project Grant/Subvention Projet
**Committee / Comité:**	Randomized Controlled Trials 2/Essais contrôlés randomisés 2
**Title / Titre:**	Examining a digital health approach for advancing schizophrenia illness self-management and provider engagement

Budget Recommendation/Recommandation budgétaire:

•$800/month for 28 months for 80 participants seems like a very high per user cost ($280 or $47/month) compared to other technology platforms I’m familiar with. It's not clear whether development costs are included.

Please indicate your appraisal of the integration of sex as a biological variable as a strength, weakness, or not applicable to the proposal./Prière de sélectionner une option pour donner votre évaluation de l'intégration du sexe comme variable biologique en tant que point fort ou point faible de la proposition, ou en tant qu'élément non applicable à la proposition.

○ **Strength/Point fort**
○ **Weakness/Point faible**
◉ **Not applicable/Non applicable**

Please indicate your appraisal of the integration of gender as a socio-cultural determinant of health as a strength, weakness, or not applicable to the proposal./Prière de sélectionner une option pour donner votre évaluation de l'intégration du genre comme déterminant socioculturel de la santé en tant que point fort ou point faible de la proposition, ou en tant qu'élément non applicable à la proposition.

◉ **Strength/Point fort**
○ **Weakness/Point faible**
○ **Not applicable/Non applicable**

**Review Type / Type d'évaluation:**	Reviewer 1 / Évaluateur 1
**Name of Applicant / Nom du chercheur:**	Kidd, Sean
**Application No. / Numéro de demande:**	426889
**Agency / Agence:**	CIHR/IRSC
**Competition / Concours:**	Project Grant/Subvention Projet
**Committee / Comité:**	Randomized Controlled Trials 2/Essais contrôlés randomisés 2
**Title / Titre:**	Examining a digital health approach for advancing schizophrenia illness self-management and provider engagement

Sex and/or Gender Considerations/Notions de sexe et/ou de genre:

Outcomes will be stratified by gender (female, male, transgender, non-binary, other)

**Review Type / Type d'évaluation:**	Reviewer 2 / Évaluateur 2
**Name of Applicant / Nom du chercheur:**	Kidd, Sean
**Application No. / Numéro de demande:**	426889
**Agency / Agence:**	CIHR/IRSC
**Competition / Concours:**	Project Grant/Subvention Projet
**Committee / Comité:**	Randomized Controlled Trials 2/Essais contrôlés randomisés 2
**Title / Titre:**	Examining a digital health approach for advancing schizophrenia illness self-management and provider engagement

Adjudication Criteria/Critères de sélection

Significance and Impact of the Research/Importance et impact de la recherche: 4.5

Approaches and Methods/Approches et méthodes: 4.5

Expertise, Experience and Resources/Expertise, expérience et ressources: 4.2

Top/Bottom Selection/Groupe supérieur/inférieur

- (●) **Top/Groupe supérieur**
- ( ) **Bottom/Groupe inférieur**

**Review Type / Type d'évaluation:**	Reviewer 2 / Évaluateur 2
**Name of Applicant / Nom du chercheur:**	Kidd, Sean
**Application No. / Numéro de demande:**	426889
**Agency / Agence:**	CIHR/IRSC
**Competition / Concours:**	Project Grant/Subvention Projet
**Committee / Comité:**	Randomized Controlled Trials 2/Essais contrôlés randomisés 2
**Title / Titre:**	Examining a digital health approach for advancing schizophrenia illness self-management and provider engagement

Summary of Application/Résumé de la demande:

This application is from a mid-career investigator from the Centre for Addition and Mental Health in Toronto. This single-blind feasibility study focuses on poor outcomes in schizophrenia due to poor adherence, limited community-based supports, and limited efforts to foster illness self-management. The investigators developed a digital health platform called Apps4Independent that addresses multiple issues. This feasibility study will evaluate typical feasibility metrics and describe efficacy endpoints over 6 months.

**Review Type / Type d'évaluation:**	Reviewer 2 / Évaluateur 2
**Name of Applicant / Nom du chercheur:**	Kidd, Sean
**Application No. / Numéro de demande:**	426889
**Agency / Agence:**	CIHR/IRSC
**Competition / Concours:**	Project Grant/Subvention Projet
**Committee / Comité:**	Randomized Controlled Trials 2/Essais contrôlés randomisés 2
**Title / Titre:**	Examining a digital health approach for advancing schizophrenia illness self-management and provider engagement

Strengths and Weaknesses/Forces et faiblesses:

Strengths:

- Well-designed and well-written, convincing proposal
- Well-described previous work leading up to this application
- Partnerships with the Centre for Addition and Mental Health as well as MEMOTEXT, a health technology company
- Excellent discussion of how this intervention differs from others under evaluation
- Intervention does not require a data plan – important consideration
- Use of passively collected data that will estimate sleep and activity – could be important
- Provider dashboard is an important aspect that may improve provider adoption of the intervention
- Nice pilot work to develop the app
- Cognisant of potential adverse effects and privacy considerations

Weaknesses:

- Would benefit from a conceptual model
- How is moderation of the peer-peer engagement platform achieved?
- It looks like the analysis will evaluate the treatment effect through generalized linear models – should not be performed in a feasibility study

Study Team:

- Appropriate with inclusion of a biostatistician
- PI – PA on a SSHRC grant, 66 publications

**Review Type / Type d'évaluation:**	Reviewer 2 / Évaluateur 2
**Name of Applicant / Nom du chercheur:**	Kidd, Sean
**Application No. / Numéro de demande:**	426889
**Agency / Agence:**	CIHR/IRSC
**Competition / Concours:**	Project Grant/Subvention Projet
**Committee / Comité:**	Randomized Controlled Trials 2/Essais contrôlés randomisés 2
**Title / Titre:**	Examining a digital health approach for advancing schizophrenia illness self-management and provider engagement

Budget Recommendation/Recommandation budgétaire:

No concerns

**Review Type / Type d'évaluation:**	Reviewer 2 / Évaluateur 2
**Name of Applicant / Nom du chercheur:**	Kidd, Sean
**Application No. / Numéro de demande:**	426889
**Agency / Agence:**	CIHR/IRSC
**Competition / Concours:**	Project Grant/Subvention Projet
**Committee / Comité:**	Randomized Controlled Trials 2/Essais contrôlés randomisés 2
**Title / Titre:**	Examining a digital health approach for advancing schizophrenia illness self-management and provider engagement

Please indicate your appraisal of the integration of sex as a biological variable as a strength, weakness, or not applicable to the proposal./Prière de sélectionner une option pour donner votre évaluation de l'intégration du sexe comme variable biologique en tant que point fort ou point faible de la proposition, ou en tant qu'élément non applicable à la proposition.

- ◉ **Strength/Point fort**
- ○ **Weakness/Point faible**
- ○ **Not applicable/Non applicable**

Please indicate your appraisal of the integration of gender as a socio-cultural determinant of health as a strength, weakness, or not applicable to the proposal./Prière de sélectionner une option pour donner votre évaluation de l'intégration du genre comme déterminant socioculturel de la santé en tant que point fort ou point faible de la proposition, ou en tant qu'élément non applicable à la proposition.

- ◉ **Strength/Point fort**
- ○ **Weakness/Point faible**
- ○ **Not applicable/Non applicable**

**Review Type / Type d'évaluation:**	Reviewer 2 / Évaluateur 2
**Name of Applicant / Nom du chercheur:**	Kidd, Sean
**Application No. / Numéro de demande:**	426889
**Agency / Agence:**	CIHR/IRSC
**Competition / Concours:**	Project Grant/Subvention Projet
**Committee / Comité:**	Randomized Controlled Trials 2/Essais contrôlés randomisés 2
**Title / Titre:**	Examining a digital health approach for advancing schizophrenia illness self-management and provider engagement

Sex and/or Gender Considerations/Notions de sexe et/ou de genre:

No concerns

**Review Type / Type d'évaluation:**	Reviewer 3 / Évaluateur 3
**Name of Applicant / Nom du chercheur:**	Kidd, Sean
**Application No. / Numéro de demande:**	426889
**Agency / Agence:**	CIHR/IRSC
**Competition / Concours:**	Project Grant/Subvention Projet
**Committee / Comité:**	Randomized Controlled Trials 2/Essais contrôlés randomisés 2
**Title / Titre:**	Examining a digital health approach for advancing schizophrenia illness self-management and provider engagement

Adjudication Criteria/Critères de sélection

Significance and Impact of the Research/Importance et impact de la recherche: 4.2

Approaches and Methods/Approches et méthodes: 3.2

Expertise, Experience and Resources/Expertise, expérience et ressources: 3.8

Top/Bottom Selection/Groupe supérieur/inférieur

- ○ **Top/Groupe supérieur**
- ◉ **Bottom/Groupe inférieur**

**Review Type / Type d'évaluation:**	Reviewer 3 / Évaluateur 3
**Name of Applicant / Nom du chercheur:**	Kidd, Sean
**Application No. / Numéro de demande:**	426889
**Agency / Agence:**	CIHR/IRSC
**Competition / Concours:**	Project Grant/Subvention Projet
**Committee / Comité:**	Randomized Controlled Trials 2/Essais contrôlés randomisés 2
**Title / Titre:**	Examining a digital health approach for advancing schizophrenia illness self-management and provider engagement

Summary of Application/Résumé de la demande:

This is a feasibility trial of a digital self-management application (App4Independence -- A4i) for patients with schizophrenia.

**Review Type / Type d'évaluation:**	Reviewer 3 / Évaluateur 3
**Name of Applicant / Nom du chercheur:**	Kidd, Sean
**Application No. / Numéro de demande:**	426889
**Agency / Agence:**	CIHR/IRSC
**Competition / Concours:**	Project Grant/Subvention Projet
**Committee / Comité:**	Randomized Controlled Trials 2/Essais contrôlés randomisés 2
**Title / Titre:**	Examining a digital health approach for advancing schizophrenia illness self-management and provider engagement

Strengths and Weaknesses/Forces et faiblesses:

Strengths

The applicants are trying to study an important but difficult to study population.

A number of appropriate feasibility outcomes are described.

Weaknesses

Is the randomization really to be stratified by gender or was sex meant?

Regarding the eligibility criteria, it is helpful to consider the inclusion criteria as define the disease of interest and exclusion criteria are reasons trial participation is not possible. With that in mind, inclusion criteria 3 and 4 are more appropriate as exclusion, suitably re-worded.

Additional detail regarding follow-up data collection is needed. Presumably, app-related data is automatic. The other outcome data needs to be collected from patients. When are these assessments done and how?

Although unnecessary for a feasibility trial, the sample size does make reference to detecting an effect on outcome. This outcome is not specified.

The analysis devotes very little attention to the feasibility assessment and instead, appears, to focus on the outcome analyses that would be done in the efficacy trial. Even then, there are some issues present. For example, the proposal states that generalized linear models will be used with a 6 month change score as the response. Assuming that the change score refers to a continuous measure, the model is simply a linear model. Furthermore, although that analysis correctly adjusts for baseline, there is no need to use change as the response in the adjusted model. The appropriateness of imputation for outcomes is also questionable. If I read it correctly, one sensitivity analysis will use psychiatrist contacts as an adjusting variable. I assume this means post-randomization. This is an invalid covariate to adjust for because it is post-randomization.

There is no point to sub-group analyses in the feasibility trial.

**Review Type / Type d'évaluation:**	Reviewer 3 / Évaluateur 3
**Name of Applicant / Nom du chercheur:**	Kidd, Sean
**Application No. / Numéro de demande:**	426889
**Agency / Agence:**	CIHR/IRSC
**Competition / Concours:**	Project Grant/Subvention Projet
**Committee / Comité:**	Randomized Controlled Trials 2/Essais contrôlés randomisés 2
**Title / Titre:**	Examining a digital health approach for advancing schizophrenia illness self-management and provider engagement

Budget Recommendation/Recommandation budgétaire:

Acceptable

**Review Type / Type d'évaluation:**	Reviewer 3 / Évaluateur 3
**Name of Applicant / Nom du chercheur:**	Kidd, Sean
**Application No. / Numéro de demande:**	426889
**Agency / Agence:**	CIHR/IRSC
**Competition / Concours:**	Project Grant/Subvention Projet
**Committee / Comité:**	Randomized Controlled Trials 2/Essais contrôlés randomisés 2
**Title / Titre:**	Examining a digital health approach for advancing schizophrenia illness self-management and provider engagement

Please indicate your appraisal of the integration of sex as a biological variable as a strength, weakness, or not applicable to the proposal./Prière de sélectionner une option pour donner votre évaluation de l'intégration du sexe comme variable biologique en tant que point fort ou point faible de la proposition, ou en tant qu'élément non applicable à la proposition.

○ **Strength/Point fort**
○ **Weakness/Point faible**
◉ **Not applicable/Non applicable**

Please indicate your appraisal of the integration of gender as a socio-cultural determinant of health as a strength, weakness, or not applicable to the proposal./Prière de sélectionner une option pour donner votre évaluation de l'intégration du genre comme déterminant socioculturel de la santé en tant que point fort ou point faible de la proposition, ou en tant qu'élément non applicable à la proposition.

○ **Strength/Point fort**
○ **Weakness/Point faible**
◉ **Not applicable/Non applicable**

**Review Type / Type d'évaluation:**	Reviewer 3 / Évaluateur 3
**Name of Applicant / Nom du chercheur:**	Kidd, Sean
**Application No. / Numéro de demande:**	426889
**Agency / Agence:**	CIHR/IRSC
**Competition / Concours:**	Project Grant/Subvention Projet
**Committee / Comité:**	Randomized Controlled Trials 2/Essais contrôlés randomisés 2
**Title / Titre:**	Examining a digital health approach for advancing schizophrenia illness self-management and provider engagement

Sex and/or Gender Considerations/Notions de sexe et/ou de genre:

Although the proposal discusses this, at a feasibility stage there is little to be done.

Review Type/Type d'évaluation: SO Notes /Notes de l'agent scientifique
Name of Applicant/Nom du chercheur: Kidd, Sean Andrew
Application No./Numéro de demande: 426889
Agency/Agence: CIHR/IRSC
Competition/Concours: 2019-09-11 Project Grant/Subvention Projet
Committee/Comité: Randomized Controlled Trials 2/Essais contrôlés randomisés 2
Title/Titre: Examining a digital health approach for advancing schizophrenia illness self-management and provider engagement

---

Assessment/Évaluation:

Strengths:

-The team is strong and multidisciplinary
-NPA is an expert in this field with a good publication track record.
-The intervention addresses multiple domains, including social isolation, medical aspects, self-management, wellness goals, etc.
-The intervention (app) is very well developed and has been beta-tested
-The study appears feasible
-Clinical outcomes are relevant
-Safety and ethical issues have been adequately addressed
-Biostatistician will be blinded
-This is a feasibility study
-Gender was addressed.

Weaknesses:

-is there a way to blind the participants to the intervention in order to increase adherence of the control group who may lose motivation to continue participation?
-recruitment feasibility has not been adequately addressed – how many sites, how many eligible patients
-peer-peer platform is a strength but there is no discussion on how abuse of the platform would be monitored and dealt with
-it is somewhat unclear how outcomes will be collected from patients
-there was extensive discussion among committee members on the fact that the trial is a feasibility study, and while feasibility outcomes are adequately described, a large part of the analysis plan tries to address efficacy

Budget:
-A quotation for the cost of the app should be provided.
